# Quantitative bone single photon emission computed tomography analysis of the effects of duration of bisphosphonate administration on the parietal bone

**DOI:** 10.1038/s41598-020-74335-y

**Published:** 2020-10-15

**Authors:** Hironobu Hata, Tomoka Kitao, Jun Sato, Takuya Asaka, Kenji Imamachi, Masaaki Miyakoshi, Kenji Hirata, Keiichi Magota, Yamato Munakata, Tohru Shiga, Yutaka Yamazaki, Yoshimasa Kitagawa

**Affiliations:** 1 Department of Dentistry and Oral Surgery, Hokkaido Cancer Center, 3-54, Kikusui4-Jyo 2-Tyoume, Shiroishi-Ku, Sapporo, Japan; 2 Department of Radiology, Hokkaido Medical Center, 1-1, Yamanote5-Jyo 7-Tyoume, Nishi-ku, Sapporo, Japan; 3 Oral Diagnosis and Medicine, Department of Oral Pathobiological Science, Faculty of Dental Medicine, Hokkaido University, Nishi 7-Tyoume Kita13-Jyo, Kita-Ku, Sapporo, Japan; 4 Department of Maxillofacial Radiology, Graduate School of Medical and Dental Sciences, Kagoshima University, 1-35-8, Sakuragaoka, Kagoshima Japan; 5 Department of Diagnostic Imaging, Graduate School of Medicine, Hokkaido University, Nishi 7-Tyoume Kita15-Jyo, Kita-Ku, Sapporo, Japan; 6 Advanced Clinical Research Center, Fukushima Global Medical Science Center, Fukushima Medical University, 1, Hikarigaoka, Fukushima, Japan; 7 Gerodontology, Department of Oral Health Science, Faculty of Dental Medicine, Hokkaido University, Nishi 7-Tyoume Kita13-Jyo, Kita-Ku, Sapporo, Japan

**Keywords:** Health care, Medical imaging

## Abstract

Effects of long-term bisphosphonate (BP) administration on the metabolism of healthy bone and the concomitant changes in imaging are unclear. Hence, we aimed to retrospectively investigate the effects of long-term BP administration on the intact parietal bone using the standardised uptake value (SUV) derived from single photon emission computed tomography (SPECT). We enrolled 29 patients who had odontogenic infection, osteoporosis, bone metastasis cancer, or rheumatoid arthritis, and classified them into BP-naïve: A (14 patients) and BP-treated: B, < 4 years (7 patients) and C, ≥ 4 years (8 patients) groups. We measured the maximum bilateral SUV (SUVmax) of the parietal bone using quantitative bone SPECT software. There were significant differences in the duration of BP administration and SUVmax of the parietal bone among the diseases (*P* < 0.0001 and *P* = 0.0086, respectively). There was a positive correlation between the duration of BP administration and SUVmax of the parietal bone (*r*_*s*_ = 0.65, *P* = 0.0002). The SUVmax was significantly different between A and B (*P* = 0.02) and between A and C (*P* = 0.0024) groups. This is the first report on the correlation between long-term BP administration and the SUVmax of the parietal bone using the quantitative bone SPECT analysis.

## Introduction

Antiresorptive drugs are the main type of drugs used to treat osteoporosis. In 2015, the sales revenue of antiresorptive drugs was approximately 109 million USD, which was more than 73% of the revenue of all drugs used to treat osteoporosis^1^. Bisphosphonates (BPs) inhibit bone resorption by preventing the development of osteoclasts via apoptosis induction^2^. The inhibition of bone resorption accompanied by unabated modelling-based bone formation results in the net bone gain^3–6^. Increased cumulative doses and long-term BP treatment are the most important risk factors for the development of BP-related osteonecrosis of the jaw (BRONJ)^7^, which has long been a problem in the medical and dental fields with no established preventive or therapeutic strategies. Bone resorption inhibitors are used for long periods without monitoring their effects or adjusting their dosage after the initiation of treatment. Recent advances in equipment for imaging and software for single photon emission computed tomography (SPECT) have enabled quantitative evaluation of standardised uptake values (SUVs) such as SUVmax, which is the maximum per pixel accumulation intensity of Tc scintigraphic preparations in a bone lesion site.

Quantitative evaluation and monitoring of bone lesions using objective indicators can provide clear diagnostic criteria for bone lesions and valuable information to treat lesions, which has been difficult to achieve so far. There are no reports of the effects of long-term BP administration on the metabolism of the parietal bone (normal bone) or the nature of changes monitored by bone SPECT.

The development of quantitative analysis software has enabled SUV analyses using SPECT^8^. We used the parietal bone of the cranial bone as a lesion control site in a previous quantitative SPECT study of osteomyelitis of the jaw bone^9^. The data of 15 patients enrolled in the study revealed that the SUV of the parietal bone increased with the BP administration period, and we hypothesised that the parietal bone may reflect the effect of systemic bone metabolism by BP. Therefore, we conducted an additional examination of the parietal bone of 29 patients, including 14 BP-naïve patients.

The objectives of this study were to compare whether there was a difference in the accumulation intensity of technetium-99 m (^99m^Tc) in the parietal bone between patients receiving BPs and BP-naïve patients by bone SPECT scintigraphy and further to investigate the effects of the duration of BP administration on the parietal bone.

## Results

### Maximum standardised uptake values of the parietal bone

**Figure 1:**
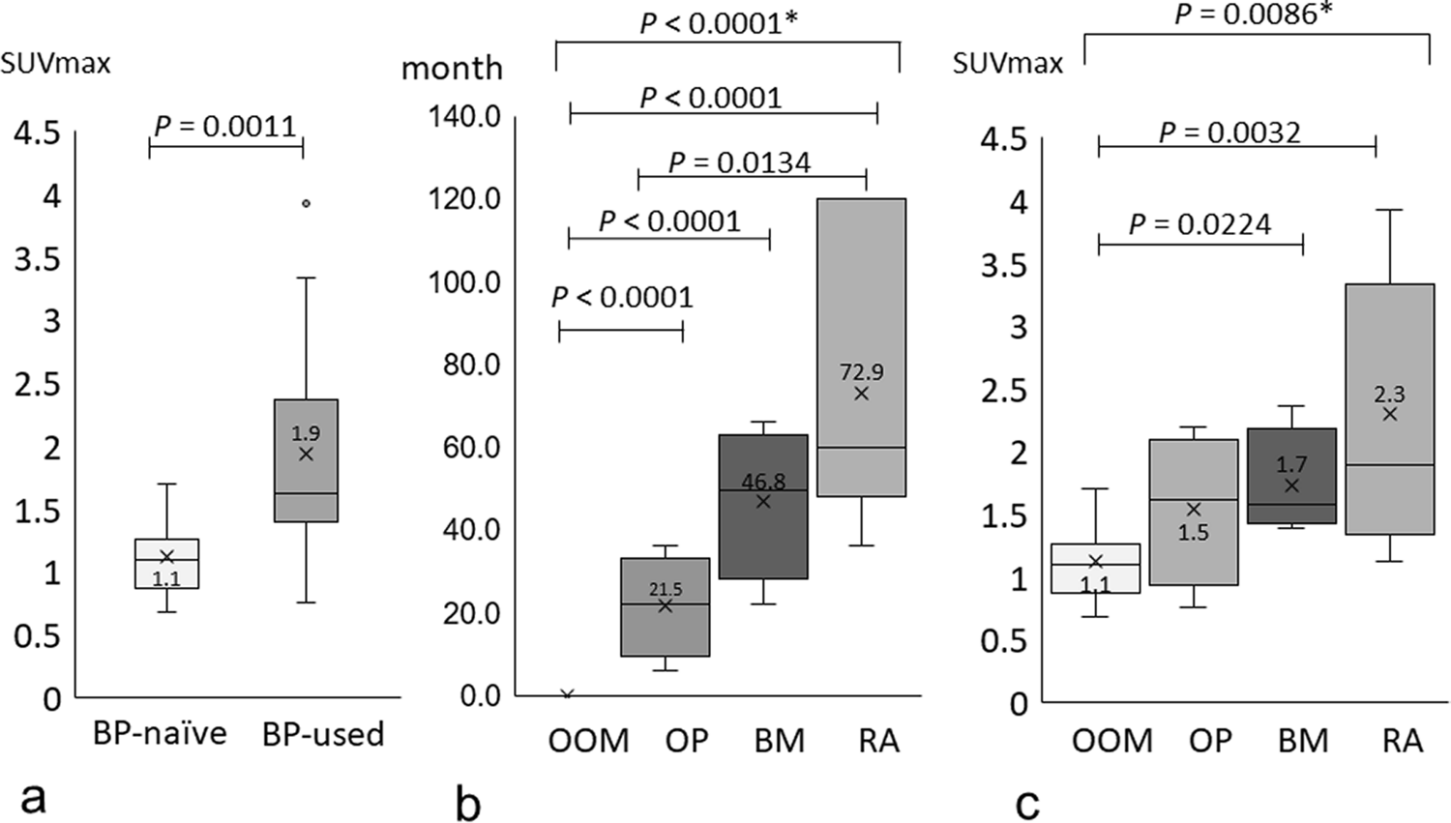
Differences in SUVmax of the parietal bone between BP-naive and BP-used patients and different duration of bisphosphonate administration and SUVmax of the parietal bone in patients with odontogenic osteomyelitis (OOM), osteoporosis (OP), bone metastasis (BM), and rheumatoid arthritis (RA). The box-and-whisker plot of the SUVmax of the parietal bone for BP naïve and BP used patients. There was a significant difference in the SUVmax between the two groups (P = 0.0011). (**a**) The number of patients with each disease was as follows: OOM = 14, OP = 4, BM = 4, and RA = 7. (**b**) The box-and-whisker plot of the duration of bisphosphonate administration versus disease. There was a significant difference in the duration of bisphosphonate administration among the diseases (*P* < 0.0001). (**b**) The box-and-whisker plot of the SUVmax of the parietal bone versus disease. There was a significant difference in the mean SUVmax of the parietal bone among the diseases (*P* = 0.0086). (**c**) When the duration of BP administration was tested in pairs between each primary disease, there was significant difference between patients with OOM and other diseases treated with bisphosphonate (*P* < 0.0001 for all pairs), but there was a significant difference between patients with OP and RA (*P* = 0.0134) (**b**). When the SUVmax was tested in pairs between each primary disease, there was significant difference between patients with OOM and patients with BM and RA (*P* = 0.0224 and *P* = 0.0032, respectively) (**c**). Kruskal–Wallis test was used to analyse data indicated with asterisks, whereas the others were tested for significance using Mann–Whitney U test.

Table 1 shows the bilateral and mean (maximum SUV) SUVmax values of the parietal bone of BP-naïve patients with odontogenic osteomyelitis of the jaw (OOM). There was no laterality in the bilateral parietal bone SUVmax values, and the overall mean SUVmax ± standard deviation (SD) of the 14 BP-naïve OOM was 1.12 ± 0.30 (range 0.68–1.70); we considered these data as baseline data for this study. Only two female patients (Pt. 9 and 10) showed distant values (the SUVmax of both was 1.70). However, there was no significant sex-related difference in the mean SUVmax of BP-naïve patients (*P* = 0.33, Mann–Whitney U test). Table 2 shows the bilateral and mean SUVmax values of the parietal bone of patients with BRONJ who received BPs for long periods. Bisphosphonates has been used to treat osteoporosis (OP) to improve bone density and bone metastatic cancer (BM) to prevent skeletal bone-related adverse events. It has also been used to prevent corticosteroid-induced osteoporosis in rheumatoid arthritis (RA), nephrotic syndrome (NPS), and other long-term systemic corticosteroid-requiring diseases. There was no laterality in the bilateral parietal bone SUVmax values, and by calculating the mean SUVmax of the left and right parietal bones, the patient-specific SUVmax of the intact bone region was more robustly obtained. The overall mean SUVmax ± SD for the patients with BRONJ was 1.94 ± 0.88 (range 0.75–3.92), which was approximately 1.7 times the mean SUVmax of the BP-naïve patients with OOM, and it showed a significant difference (*P* = 0.0011, Mann–Whitney U test) (Fig. 1a).

**Table 1:** Characteristics of patients with odontogenic osteomyelitis.

No	Sex	Age (years)	OMJ	Right parietal bone (SUVmax)	Left parietal bone (SUVmax)	Mean (SUVmax)
1	M	60	Mandible	0.99	0.77	0.88
2	M	65	Mandible	0.68	0.68	0.68
3	M	66	Mandible	0.97	1.21	1.09
4	M	68	Mandible	1.18	1.03	1.11
5	M	69	Maxilla	0.70	0.97	0.84
6	M	71	Mandible	1.13	1.51	1.32
7	M	74	Mandible	1.11	1.06	1.09
8	M	50	Mandible	1.15	1.32	1.24
9	F	67	Mandible	1.65	1.75	1.70
10	F	58	Mandible	1.70	1.69	1.70
11	F	70	Mandible	1.20	1.09	1.15
12	F	72	Mandible	0.77	0.73	0.75
13	F	75	Mandible	0.97	1.01	0.99
14	F	53	Mandible	1.01	1.22	1.12
Mean		65.57		1.09	1.15	1.12
SD		7.64		0.30	0.33	0.30

*OMJ* osteomyelitis of the jaw, *SD* standard deviation; all patients were BP-naïve, *SUVmax* maximum standardised uptake value.

**Table 2:** Characteristics of patients with BRONJ who used bisphosphonates.

No	Sex	Age (years)	BRONJ stage	Jaw	Primary disease	Bisphosphonate	Administration route	Medication period (months)	Right parietal bone (SUVmax)	Left parietal bone (SUVmax)	Mean (SUVmax)
15	F	90	2	Mandible	OP	Risedronate	IO	6	1.70	1.85	1.77
16	F	79	3	Mandible	OP	Risedronate	IO	24	1.51	1.39	1.45
17	F	81	2	Mandible	OP	Risedronate	IO	36	0.81	0.69	0.75
18	F	79	2	Mandible	OP	Alendronate	IO	20	1.96	2.43	2.20
19	M	75	3	Mandible	BM (PC)	Zoledronate	IV	22	1.42	1.80	1.61
20	F	67	3	Maxilla	BM (BC)	Zoledronate	IV	66	1.60	1.50	1.55
21	F	58	2	Mandible	BM (BC)	Zoledronate	IV	53	1.48	1.30	1.39
22	F	76	2	Mandible	BM (BC)	Zoledronate	IV	46	2.25	2.49	2.37
23	F	78	3	Maxilla	NPS	Alendronate	IO	66	1.47	1.38	1.43
24	M	80	2	Mandible	RA	Alendronate	IO	48	1.14	1.10	1.12
25	F	78	3	Mandible	RA	Risedronate	IO	36	1.30	1.39	1.34
26	F	63	2	Mandible	RA	Alendronate	IO	60	3.03	2.97	3.00
27	F	76	2	Mandible	RA	Risedronate	IO	60	3.77	2.89	3.33
28	F	77	2	Mandible	RA	Alendronate	IO	120	1.99	1.79	1.89
29	F	66	2	Maxilla	RA	Risedronate	IO	120	4.08	3.77	3.92
Mean		74.87						52.20	1.97	1.92	1.94
SD		8.11						32.90	0.95	0.83	0.88

*SD* standard deviation, *BRONJ* bisphosphonate-related osteonecrosis of the jaw, *BC* breast cancer, *BM* bone metastasis, *DM* diabetes mellitus, *IV* intravenous, *IO* intraoral, *NPS* nephrotic syndrome, *OP* osteoporosis, *PC* prostate cancer, *RA* rheumatoid arthritis.

### Differences in the duration of BP administration and SUVmax for various primary diseases

The correlation between the duration of BP administration and each primary disease (Fig. 1b) was compared with that between the SUVmax of the parietal bone and each primary disease (Fig. 1c).

**Figure 2:**
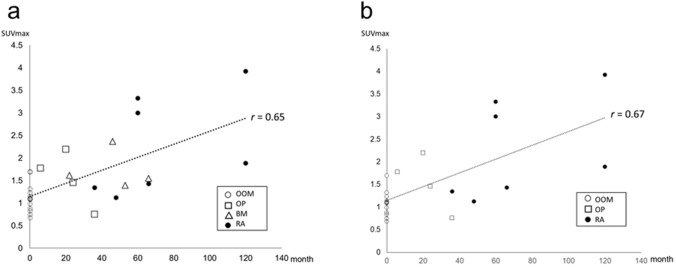
Changes in the SUVmax of the parietal bone with the duration of bisphosphonate administration. (**a**) The scatter plot of SUVmax of the parietal bone versus duration of bisphosphonate administration for all diseases showed a positive correlation between the two variates (*r*_*s*_ = 0.65, *P* = 0.0002). The correlation line equation and the coefficient of determination were: Y = 0.014 X + 1.154, R^2^ = 0.427. (**b**) The scatter plot of the SUVmax of the parietal bone versus duration of bisphosphonate administration for all diseases, after excluding four patients with bone metastasis treated with intravenous bisphosphonate, showed a positive correlation between the two variates (*r*_*s*_ = 0.62, *P* = 0.0009). The correlation line equation and the coefficient of determination were: Y = 0.0152 X + 1.150, R^2^ = 0.459.

There were significant differences in the duration of BP administration and the SUVmax of the parietal bone among the diseases (*P* < 0.0001 and *P* = 0.0086, Kruskal–Wallis test), respectively. When the duration of BP administration was tested in pairs between each primary disease, there was a significant difference between patients with OP (mean 21.5 months) and patients with RA (mean 72.9 months) when the patient with OOM (BP-naïve) was excluded (*P* = 0.0134 Mann–Whitney U test) (Fig. 1b). When the SUVmax was tested in pairs between each primary disease, there was a significant difference between patients with OOM (mean 1.1) and patients with BM (mean 1.7) and patients with RA (mean 2.3), respectively (*P* = 0.0224 and *P* = 0.0032 Mann–Whitney U test) (Fig. 1c). The duration (months) ± SD/SUVmax ± SD values for the different primary diseases were as follows: OOM (0.00 ± 0.00/1.12 ± 0.30), OP (21.50 ± 12.37/1.59 ± 0.56), BM (46.75 ± 18.46/1.73 ± 0.38), and RA (72.86 ± 33.66/2.13 ± 0.95). A moderate positive correlation was observed between the duration of BP administration and the SUVmax in the parietal bone of all 29 patients (*r*_*s*_ = 0.65, *P* = 0.0002 Spearman’s rank correlation coefficient) (Fig. 2a). Four patients with BM, who were intravenously administered zoledronate, were excluded from the analysis; this resulted in a slight increase in the positive correlation between the duration of oral BP administration and the SUVmax of the parietal bone (*r*_*s*_ = 0.62, *P* = 0.0009 Spearman’s rank correlation coefficient) (Fig. 2b). A positive correlation between the duration of BP administration and SUVmax of the parietal bone suggests that longer BP administration increases bone formation.

### Comparison among three groups with a cut-off BP administration period of 4 years

**Figure 3:**
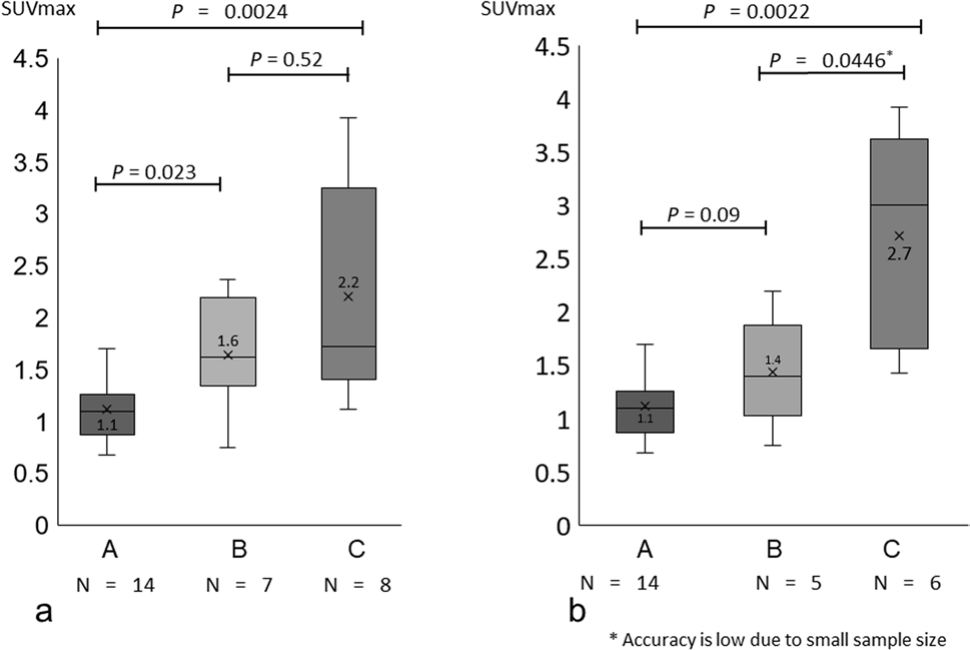
Changes in the SUVmax of the parietal bone with the duration of bisphosphonate administration, including and excluding patients with bone metastasis. (**a**) The box-and-whisker plot of the SUVmax of the parietal bone of three groups of patients categorised according to the duration of bisphosphonate administration: A, BP naïve (14 patients), B: < 4 years (7 patients), C: ≥ 4 years (8 patients). There was a significant difference between Group A and Group B (*P* < 0.05), but no significant difference was found between Group B and Group C (*P* = 0.52). (**b**) The box-and-whisker plot of the SUVmax of the parietal bone in all patients classified into three groups after excluding four patients with bone metastasis treated with intravenous bisphosphonate. There was no significant difference between A and B (*P* = 0.09). Although there was a significant difference between B and C (*P* < 0.05), its accuracy was considered to be low because of the small sample size of Group C.

As described in detail later in the Discussion, as the risk of BRONJ increases four-fold when the administration period of oral BPs exceeds 4 years, the patients were classified into the following three groups: A, BP-naïve (14 patients); B, BP treatment for less than 4 years (7 patients); and C, BP treatment for 4 years or more (8 patients) (Table 2). Their duration (months) ± SD/SUVmax ± SD values were as follows: A, 0.00 ± 0.00/1.12 ± 0.30; B, 27.14 ± 13.21/1.64 ± 0.51; and C, 74.13 ± 28.96/2.09 ± 0.87. The differences in the SUVmax values were not significant between B and C (*P* = 0.45), but they were significant between A and B (*P* = 0.023, Mann–Whitney U test) and between A and C (*P* = 0.0024, Mann–Whitney U test) (Fig. 3(a)). To distinguish oral BPs (low-dose BPs) from intravenous BPs (high-dose BPs), four BM patients who were treated with zoledronate intravenously were excluded from the analysis. This exclusion resulted in significant differences between B and C (*P* = 0.0446, Mann–Whitney U test), but not between A and B (*P* = 0.09, Mann–Whitney U test). However, as the exclusion of the four BM patients also decreased the number of patients in group C by 2, the comparison between B and C was considered less accurate because of the small sample size (6 patients) (Fig. 3b). These results suggest that low-dose BP patients and high-dose BP patients should be discussed separately in the future.

## Discussion

Our data revealed a positive correlation between the SUVmax of the parietal bone and the duration of BP administration. It presented a moderate correlation coefficient (r_s_ = 0.65 and 0.62), but because of the small number of cases, the coefficient of determination (R^2^ = 0.43 and 0.46) was low and the linear correlation was not high. Furthermore, there were significant differences in the SUVmax values among the different primary diseases, OOM, OP, BM, and RA; therefore, we consider that patients with each primary disease should not be regarded as a homogenous population, they only reflect the duration of BP administration. One patient with NPS was assigned to the RA group because low-dose BPs was used to prevent steroid-induced OP, and high-dose BPs are not used in such patients, as in patients with BM. In all cases, there was a significant increase in the SUVmax of the BP-treated groups (B and C) compared with that of the BP-naïve group (A) (A vs BPs = 0.023, A vs C: *P* = 0.0024). Furthermore, the increase in the SUVmax for the ≥ 4-year group (C) relative to the < 4-year group (B) was not significant (B vs C: *P* = 0.52). In the low-dose BP patients, there was a significant increase in the SUVmax in group C compared with that in group B with the proviso that accuracy is low due to the small sample size (C vs BPs = 0.0446). According to these results of significant trend changes, it may not be appropriate to compare the BM group that used intravenous zoledronate as a high-dose BP and other groups that used oral low-dose BPs. According to the 2007 Predicting Risk of Osteonecrosis of the Jaw with Oral Bisphosphonate Exposure (PROBE) study, the risk of BRONJ from oral BP formulations has increased by approximately four times from 0.05 to 0.21 over 4 years^10^. The American Association of Oral and Maxillofacial Surgeons (AAOMS) position paper also supports this view; for patients orally administered BP for < 4 years and no other clinical risk factors, it recommends that BP need not be discontinued during oral surgery such as tooth extraction^11^. Considering the effects on bone metabolism in our BP-naïve patients and oral BP patients, we support the position paper’s proposal that 4 years could be a cut-off for BRONJ as a risk factor during BP administration. However, it is important to note that BRONJ occurred in patients who received oral BPs for less than 4 years. Indeed, the risk of BRONJ is expected to increase with longer periods of administration. Although it is not certain that the parietal bone is the most appropriate evaluation site in the whole skeletal bone, it is definitely a site that is not susceptible to odontogenic inflammation and maxillary sinusitis in our SPECT imaging range. The cranial bone consists of the ethmoid, sphenoid, frontal, parietal, temporal, and occipital bones. Anatomically, the maxillary sinusitis can spread to ethmoid sinus, sphenoid sinus, and frontal sinus that constitute the sinuses. Inflammation may also spread from the ethmoid sinus to the orbit. Based on the analysis of 1077 patients with odontogenic maxillofacial infections, it was reported that the spread of inflammation to the temporal bone was 3.2% and that osteomyelitis of the temporal bone may occur due to otitis media^12^. In addition, it has been reported that intracranial abscess^13^ and epidural abscess of odontogenic infection^14^ are extremely rare, and we consider that the risk of transmission of inflammation to the parietal and occipital bones is low; therefore, we chose the parietal bone for this study. Here, we showed that the parietal bone may be a critical site to assess the risk of BRONJ, and thus facilitate the evaluation of metabolism of normal bone based on the duration of BP administration.

Diphosphonate derivatives radiolabelled with ^99m^Tc, such as ^99m^Tc-conjugated BPs (Tc-BPs: Tc-methylene diphosphonate (Tc-MDP) and Tc-hydroxymethylene diphosphonate (Tc-HMDP), have been used for more than 40 years in nuclear medicine and recognised as bone-seeking agents with particular affinity for areas of active mineralisation or bone formation areas, implying the involvement of osteoblastic cells^15,16^. Im et al*.* demonstrated that, besides their role as inhibitors of osteoclastic bone resorption, BPs are promoters of osteoblast proliferation and maturation^17^. Our results of altered Tc-HMDP bone uptake depending on the duration of BP administration demonstrated the effect of BPs as antiresorptive drugs on remodelling-induced suppression activity or apoptosis of osteoclasts, and the relative enhancement of osteoblast activity.

The BP preparation originally developed as a therapeutic drug for osteoporosis is administered systemically, and it affects bone metabolism not only in the cranial or jaw bone but also whole skeletal bone. Only a few studies have performed functional imaging to evaluate the effects of BP administration on normal bone. Using bone scintigraphy, Ristow et al*.* showed that, after 12 months of intravenous administration of zoledronate, bone turnover slightly increased in the investigated intact bone regions, such as the frontal bone, maxilla, and mandible, but not the femur, in patients with breast cancer^18^. In a study performed in patients with prostate cancer, the mandible and femur showed significantly lower bone turnover than the frontal bone and maxilla, whereas bone turnover was slightly higher in the mandible and femur at 24 months after intravenous BP administration^19^. Neither study showed any significant difference in bone turnover in the investigated intact bone regions after 24 months of BP administration. Obayashi et al*.* initially evaluated the planar bone scintigraphic images of patients who had been treated with BPs for long periods (mean period of 4.9 years). They showed significantly higher bone uptake values (BUV) in the intact mandible and bone metabolism after long-term BP administration than those in the BP-naïve control group^20^. Furthermore, these researchers showed that bone metabolism was suppressed in the femurs of BP-treated patients with OP but was enhanced in the mandible compared with that in the control group^21^. There was no significant difference in bone metabolism between the two groups administered low-dose BPs and high-dose BPs^21^. However, although these studies used semi*-*quantitative analyses using planar images and OsiriX region of interest (ROI) tool, neither study considered the differences in the duration of BP administration. In the four aforementioned studies, the uptake level of ^99m^Tc in scintigraphy was interpreted as being synonymous with bone turnover or bone metabolic activity, although the metabolic activity was reduced in osteoclasts by BPs. Hence, we propose a more precise description of osteogenic dominancy in remodelling instead of only referring to an increase in metabolic activity due to BP administration.

In general clinical practice, high-dose BPs are intravenously administered every 4 weeks after the start of administration; however, recently published studies showed non-inferiority of 12-week versus 4-week schedules of BP administration^22^. The administration interval^23^ and the need for switching BPs^24^ are still controversial. This method of measuring and monitoring the SUVmax of SPECT of the parietal bone of patients, who are treated with BPs, highlights two essential requirements during treatment. First, in patients using bone resorption inhibitors, clinicians should assess the need for a drug holiday to allow recovery of the bone metabolic balance during the drug-holiday period when tooth extraction or any other oral surgery is required. Second, appropriate tailor-made dosage intervals and duration of BP administration should be determined. In the present study, there was an individual difference of 0.68–1.70 among BP-naïve patients, and some patients showed a high SUVmax, even those with short-term BP administration. For BRONJ risk assessment and adjustment of BP administration interval for each patient, we anticipate that the cumulative value from baseline will be more important than the SUVmax value at each time point. It is well known that the risk of developing BRONJ differs between patients receiving intravenous high-dose BPs and those receiving oral BPs, with a low bioavailability. The incidence of BRONJ in the USA was 0.8–12% with intravenous BPs and 0.0007% (0.7/100 000) person-years of exposure with oral BPs^24^. Therefore, we performed an analysis including the BM group (with high-dose BPs) and an analysis excluding the BM group. In the two groups, no significant difference was found in moderate positive correlation between the duration of BP administration and the SUVmax in the parietal bone. Although in the latter group of patients, a significant difference in the SUVmax was observed between patients treated with BPs for < 4 years and those treated with BPs for ≥ 4 years, the accuracy of comparison was deemed to be low because of the small sample size.

There were several study limitations to this study. We should not evaluate patients who had been treated with low-dose BPs orally and those who had been treated with high-dose BPs intravenously together if the number of cases is sufficient. This was an exploratory retrospective study, which was performed in a single centre. The results of this study are limited to BP-treated patients, and it is unclear whether similar results will be obtained in patients treated with another anti-resorptive drug, denosumab. Although the uptake value of the parietal bone was measured in patients grouped according to the duration of BP administration in this cross-sectional study, it was not evaluated over time in these patients. To evaluate the accumulation value for each patient, SPECT imaging will be necessary at the start of BP administration to serve as a baseline. Additionally, to increase the sample size, multi-centre studies will be required to enable harmonisation of devices.

In summary, this is the first study to compare the uptake values of ^99m^Tc-HMDP of the parietal bone of BP-naïve patients and BP-treated patients using the quantitative bone SPECT scintigraphic analysis, and our results showed a significant correlation between the duration of BP administration and the SUVmax of the parietal bone. Furthermore, we have shown for the first time that intact parietal bone can be used to assess metabolic changes in the skeletal bone following BP administration and that these changes may be used as an indicator to withdraw and adjust dosing intervals in patients administered BPs.

## Methods

### Study design

The present study was a pilot, observational, cross-sectional study. The study protocol and patient information were approved by the Hokkaido University Hospital Ethics Review Board (clinical study number 012-0111), and informed consent was waived by the ethics board owing to the retrospective nature of the study. Using the bone SPECT quantitative analysis data, we retrospectively compared the metabolic differences in the parietal bone of patients who had been treated with BPs for long periods with those who did not receive any BP (control group). All experiments were performed in accordance with relevant guidelines and regulations.

### Patient characteristics

As this was an exploratory study, we included more than 12 patients in each of the BP-naïve and BP-used groups, following the Julious’s sample size of 12 per group rules of thumb for a pilot study^25^. Fourteen patients with OOM and 15 patients with BRONJ who visited Hokkaido University Hospital’s Oral Diagnosis and Medicine Department from 1 July, 2008 to 31 September, 2014 were included in this retrospective clinical study. The OOM patient group consisted of 8 men and 6 women with the median age of 67.5 (range 50–75) years (Table 1). OOM was caused by odontogenic infections, and these patients had never been treated with BPs. The BRONJ patient group comprised patients treated in our previous study of quantitative SPECT for osteomyelitis of the jaw^9^. The group consisted of 2 men and 13 women with the median age of 74.9 (range 58–90) years. This BP-treated and BRONJ group had the following primary diseases: osteoporosis (n = 4), bone metastasis cancer (n = 4), rheumatoid arthritis (n = 6), and NPS syndrome (n = 1). The one patient with NPS was assigned to the RA group (Table 2).

### Bone SPECT and quantitative analyses

Bone SPECT was performed 4 h after an intravenous injection of 555 MBq Tc-99m hydroxymethylene diphosphonate (^99m^Tc-HMDP; Nihon Medi-Physics Co., Ltd., Tokyo, Japan) using a SPECT system (ECAM; Siemens Healthcare, Erlangen, Germany). The SPECT images were acquired with the following settings: low-energy high-resolution collimator, step-and-shoot mode with 30 s per step and 72 steps per detector, 360°, a matrix size of 128 × 128, a pixel size of 3.30 mm, and the energy window of 140 keV ± 10%. The SPECT images were reconstructed using the ordered-subset expectation maximisation (OSEM) method with 2 iterations and 12 subsets. Images were smoothened using a 3D spatial Gaussian filter of 8.4 mm at full width at half maximum (FWHM). Becquerel calibration factor (BCF), a numeric factor used to convert a pixel value into the SUV, was determined using a cylindrical phantom filled with uniform ^99m^Tc solution. BCF was determined at 12,953 Bq (counts/s); the SUV was calculated as described previously^26^:

$$ \left[ {BCF \, \left( {Bq/cps} \right) \, \times \, body \, weight \, \left( g \right) \, \times \, SPECT \, count \, density \, \left( {count/cc} \right)} \right]/[scan \, duration \, \left( s \right) \, \times \, injected \, activity \, \left( {Bq} \right) $$

**Figure 4:**
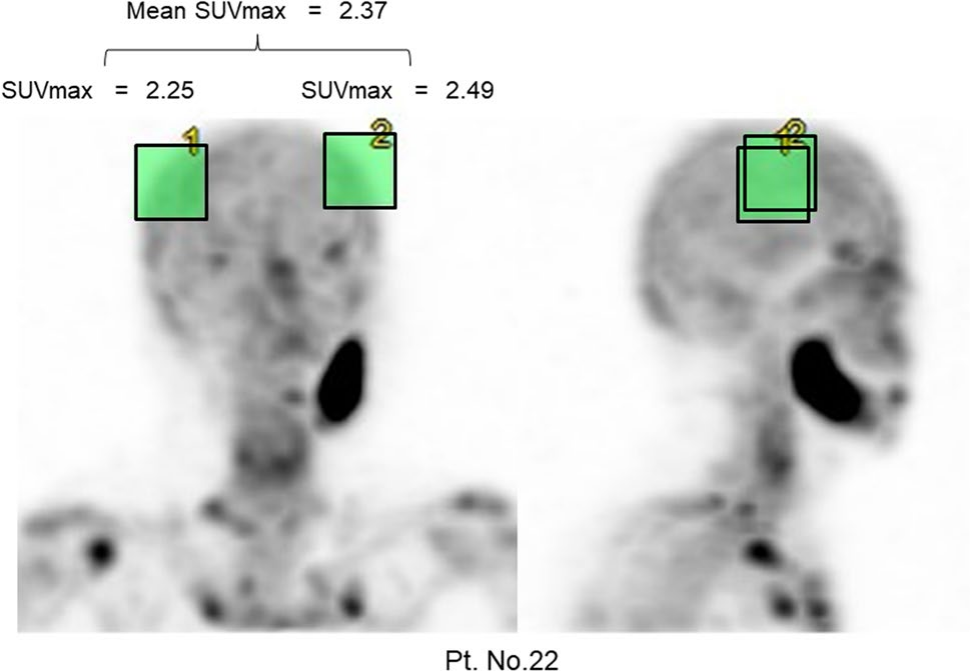
Volume-of-interest settings of bilateral parietal bone. Bilateral parietal bone SUVmax was calculated for the representative patient (No. 22) by setting the volume of interest within a 12-pixel cube (62.1 cm^3^). In all cases, the parietal bones were less susceptible to odontogenic infections and were suitable for intact bone assessment. GI-BONE, the bone SPECT quantitative analysis software, included in the medical device software package named AZE Virtual Place Hayabusa Ver.9.0 (Canon medical systems Co., Ltd., Tochigi, Japan) was used. URL: https://jp.medical.canon/products/healthcareIT/aze/virtual_place_hayabusa/.

GI-BONE, the bone SPECT quantitative analysis software, included in the medical device software package named AZE Virtual Place Hayabusa Ver.9.0 (Canon medical systems Co., Ltd., Tochigi, Japan), was used to calculate the SUVmax. The software computed the SUVmax of the parietal bone by setting the volume of interest (VOI). Each patient’s normal bone and the reproducibility of SUVmax were calculated using SPECT quantitative analysis software by setting the VOI within a 12-pixel (3.96 cm) cube (Fig. 4).

### Statistical analysis

The correlation between the duration of BP administration and SUVmax of the parietal bone for each patient was determined by calculating the Spearman’s rank correlation coefficient. Other data comparisons were made using Mann–Whitney U test or Kruskal–Wallis test. The results with *P* < 0.05 were considered statistically significant. These data were analysed using SPSS 25 (Statistical Package for the Social Sciences, IBM Corp., NY, USA) software.
